# The Cannabinoid Receptor 1 Reverse Agonist AM251 Ameliorates Radiation-Induced Cognitive Decrements

**DOI:** 10.3389/fncel.2021.668286

**Published:** 2021-06-28

**Authors:** Vipan K. Parihar, Amber Syage, Lidia Flores, Angelica Lilagan, Barrett D. Allen, Maria C. Angulo, Joseph Song, Sarah M. Smith, Rebecca J. Arechavala, Erich Giedzinski, Charles L. Limoli

**Affiliations:** Department of Radiation Oncology, University of California, Irvine, Irvine, CA, United States

**Keywords:** cranial irradiation, mood and memory deficits, AM251, neurogenesis, HMGB1

## Abstract

Despite advancements in the radiotherapeutic management of brain malignancies, resultant sequelae include persistent cognitive dysfunction in the majority of survivors. Defining the precise causes of normal tissue toxicity has proven challenging, but the use of preclinical rodent models has suggested that reductions in neurogenesis and microvascular integrity, impaired synaptic plasticity, increased inflammation, and alterations in neuronal structure are contributory if not causal. As such, strategies to reverse these persistent radiotherapy-induced neurological disorders represent an unmet medical need. AM251, a cannabinoid receptor 1 reverse agonist known to facilitate adult neurogenesis and synaptic plasticity, may help to ameliorate radiation-induced CNS impairments. To test this hypothesis, three treatment paradigms were used to evaluate the efficacy of AM251 to ameliorate radiation-induced learning and memory deficits along with disruptions in mood at 4 and 12 weeks postirradiation. Results demonstrated that acute (four weekly injections) and chronic (16 weekly injections) AM251 treatments (1 mg/kg) effectively alleviated cognitive and mood dysfunction in cranially irradiated mice. The beneficial effects of AM251 were exemplified by improved hippocampal- and cortical-dependent memory function on the novel object recognition and object in place tasks, while similar benefits on mood were shown by reductions in depressive- and anxiety-like behaviors on the forced swim test and elevated plus maze. The foregoing neurocognitive benefits were associated with significant increases in newly born (doublecortin+) neurons (1.7-fold), hippocampal neurogenesis (BrdU+/NeuN+mature neurons, 2.5-fold), and reduced expression of the inflammatory mediator HMGB (1.2-fold) in the hippocampus of irradiated mice. Collectively, these findings indicate that AM251 ameliorates the effects of clinically relevant cranial irradiation where overall neurological benefits in memory and mood coincided with increased hippocampal cell proliferation, neurogenesis, and reduced expression of proinflammatory markers.

## Introduction

Every year, more than 150,000 cancer patients in the United States receive radiotherapy for primary and metastatic brain tumors (Owonikoko et al., 2014; Makale et al., 2017). While advances in radiotherapy have greatly improved the treatment of cancer, cognitive disabilities persist in 80% of those surviving their treatments (Giovagnoli and Boiardi, 1994). Resultant neurocognitive complications involve a spectrum of associated toxicities, exhibiting highly variable time courses that include multiple cognitive domains. Deficits in learning, memory, processing speed, attention, and executive function (Roman and Sperduto, 1995; Makale et al., 2017) can manifest from months to years after irradiation at variable rates of progression and severity. While the precise molecular pathways involved in radiation-induced normal tissue toxicities remain to be elucidated, altered hippocampal neurogenesis, elevated oxidative stress, and neuroinflammation most certainly play a role (Monje and Palmer, 2003; Monje et al., 2007; Greene-Schloesser et al., 2012, 2013; Oh et al., 2013).

Oxidative and inflammatory cascades known to persist long-after irradiation (Tofilon and Fike, 2000; Greene-Schloesser et al., 2012, 2013) and the neurogenic regions harboring neural stem and progenitor cells have been shown to be exquisitely sensitive to irradiation in preclinical models (Monje et al., 2002; Mizumatsu et al., 2003). In efforts to provide some relief from the unintended neurocognitive complications arising from cranial radiotherapy, treatment plans incorporating hippocampal avoidance have become more commonplace. While such strategies have been shown to be beneficial (Gondi et al., 2014; Brown et al., 2020), a significant fraction of resultant radiation-induced deficits persist, leaving this as a largely unmet medical need. Given the paucity of effective treatment strategies for the long-term preservation of neurological health in brain cancer survivors, efforts to identify efficacious treatments able to ameliorate or prevent radiation-induced CNS toxicities remain a topical area of research.

Significant past data has pointed to the potential promise of manipulating the cannabinoid system for resolving a variety of neurological complications, albeit to date, not for the resolution of radiation-induced brain injury. Pathways involving the cannabinoid receptor 1 (CB1) mediate diverse physiologies and have long been considered potential therapeutic targets. A large body of evidence in both animal and human studies suggests that CB1 antagonism is highly effective for the treatment of obesity, metabolic disorders, and drug addiction (Gueye et al., 2016). However, the first-in-class CB1 antagonist/inverse agonist, rimonabant, though demonstrating effectiveness for obesity treatment and smoking cessation, displayed certain adverse psychiatric side effects, including anxiety and depression, resulting in its eventual withdrawal from the European market (Christensen et al., 2007; Sam et al., 2011). Interestingly, second-generation CB1 blockers now provide safer alternatives to previous and highly brain-penetrant agents for the treatment of metabolic disorders, including diabetes, obesity, and weight loss with better psychiatric tolerability (Gueye et al., 2016). Behavioral studies have also indicated the promise of AM4113 for the treatment of opioid, nicotine, marijuana, and alcohol abuse (Sink et al., 2008; Cluny et al., 2011). Furthermore, cannabidiol, a non-psychoactive component of cannabis, and selective CB1 agonist has been approved recently by the FDA for the treatment of pediatric epilepsy (Silvestro et al., 2019) and is and under clinical trials for the treatment of anxiety disorders (Silvestro et al., 2019).

Particularly relevant to the current investigation were findings showing that stimulation of CB could enhance adult neurogenesis (Hill et al., 2010). Related studies have found that the synthetic (CB1) inverse agonists AM251 and SR141716A could enhance hippocampal neurogenesis and survival of mature neurons (Hill et al., 2010; Wolf et al., 2010; Hutch and Hegg, 2016). Other work corroborates these findings by providing evidence for the neuroprotective effects of AM251 against both neurotoxic chemical insults as well as in various models of neuronal damage and neurodegenerative diseases (Shearman et al., 2003; Chen et al., 2004; Bialuk and Winnicka, 2011). The beneficial effects of AM251 have also been demonstrated in other preclinical rodent models of brain injury, where treatments were shown to ameliorate deficits observed in mood-related behaviors and memory (Bialuk and Winnicka, 2011; Balla et al., 2018). The foregoing prompted the current investigations aimed at determining whether modulating cannabinoid signaling through the use of AM251 would prove beneficial in the irradiated brain. To our knowledge, this is the first study to investigate the potential neuroprotective effects of AM251 against radiation-induced brain injury over acute and protracted postirradiation time points, after carefully selected administration regimens. Here, we report our findings detailing the neurocognitive, proneurogenic and anti-inflammatory benefits of three distinct AM251 administration regimens following cranial irradiation in mice.

## Materials and Methods

### Animals and Irradiation

All animal procedures described in this study were in accordance with NIH guidelines and approved by the University of California Irvine Institutional Animal Care and Use Committee. Wild-type male mice (C57BL/6J) 6 months of age approximate the age at which humans are at higher risk of developing glioblastoma multiforme (GBM, median age 64 years). Therefore, the older aged animal model provides a more faithful representation of the normal brain responses expected from patient cohorts afflicted with GBM and that might stand to benefit from cranial irradiation and AM251 treatment. Mice were group housed in ventilated cages maintained under standard housing conditions (20 ± 1°C; 70 ± 10% humidity; 12:12 h light-dark schedule) and provided *ad libitum* access to food (Envigo Teklad 2020x, Indianapolis, IN, United States) and water. Unique cohorts used for the 4-, 12-, and 13-week study were divided into four experimental groups (*n* = 8–12 mice per group): unirradiated receiving 1% ethanol as vehicle (0 Gy + Veh), unirradiated receiving CB1 inverse agonist AM251 (0 Gy + AM251), head-only irradiation receiving 1% ethanol (9 Gy + Veh), and head-only irradiation receiving AM251 (9 Gy + AM251). For cranial irradiation, mice were anesthetized (5% induction and 2% maintenance isoflurane, vol/vol), placed ventrally on the treatment table (XRAD 320 irradiator) for head-only irradiation delivered at a dose rate of 1.0 Gy/min (Parihar and Limoli, 2013; Acharya et al., 2016).

### AM251 Treatments

**FIGURE 1:**
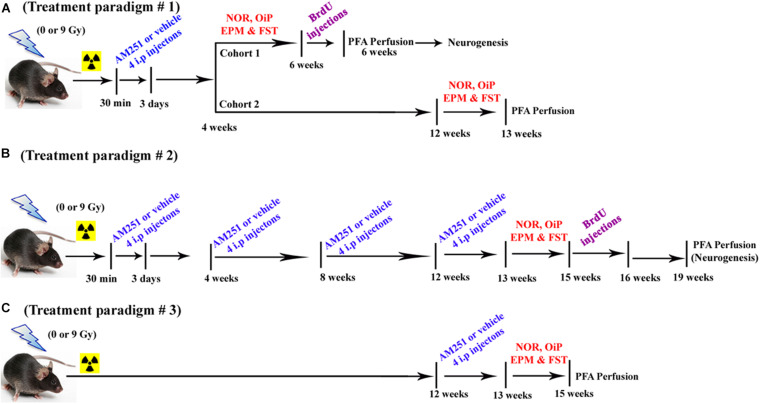
Timeline of AM251 treatments and analyses. **(A)** Short-term, **(B)** long-term, and **(C)** delayed AM251 treatment paradigms used in this study.

The various treatment paradigms implemented in this study are shown in Figure 1. For the short-term treatment arm (Figure 1A), each cohort received their first intraperitoneal (i.p.) injection of AM251 (1 mg/kg dissolved in 1% of ethyl alcohol, Sigma-Aldrich, St. Louis, MO, United States) at 30 min postirradiation and then once daily for 3 days, while mice in control groups received an equal amount of 1% of ethyl alcohol, i.p. The dose of AM251 was based on previous findings showing improvements in cognition and neurogenesis (Shearman et al., 2003; Bialuk and Winnicka, 2011). Injections of the drug were given to alternative sides of the peritoneal cavity to minimize irritation. Animals exhibited no indications of dermal or peritoneal irritation or weight loss. Following irradiation, animals in all groups were tested for changes in mood and cognition using a variety of behavioral platforms in the following sequence: novel object recognition test (NOR, for examining object recognition memory), object-in-place (OiP, for investigating spatial learning and memory), elevated plus maze test (EPM, for assessing anxiety-like behavior), and forced swim test (FST, for assessing depressive-like behavior). For the longer-term treatment arm, injections of AM251 proceeded as described above, where four i.p. injections were given every 4 weeks over a period of 12 weeks (total 16 i.p. injections in 12 weeks period; Figure 1B). Similar to short-term treatments, mice in the long-term treatment received the first injection of AM251 at 30-min postirradiation and then once daily for 3 days. Mice were given three more rounds of this regimen spaced by 4-week interval. 2 days after the last injection of AM251 (13 weeks after irradiation), animals in all groups (12/group) were tested for neurocognitive changes as before. For the delayed treatment arm, mice received four daily i.p. injections of AM251 12 weeks after irradiation (Figure 1C). Two days after the last injection of AM251, animals in each group (12/group) were tested for alterations in neurocognitive function as before.

### Behavioral Testing and Follow-Up Analyses

All behavioral testing was conducted at the times indicated in Figure 1 and always transpired after irradiation. For treatment paradigm #1 (acute AM251, four injections), behavioral testing started at week 4 or week 12 for the short- and long-term arms, respectively. For treatment paradigm #2 (chronic AM251, 16 injections), behavioral testing started at week 13 postirradiation. For treatment paradigm #3 (acute AM251, four injections—the reversal arm), behavioral testing started at week 13 postirradiation. Detailed methods and procedures regarding behavioral testing and all follow-up procedures used for immunohistochemistry and the stereological quantification of neurons are provided in the Supplementary Material.

### Statistical Analyses

All data were analyzed, and figures created with, GraphPad Prism v6.0 (GraphPad Software; La Jolla, CA, United States). All the behavioral and immunohistochemistry were analyzed using two-way ANOVA considering radiation and drug treatment (AM251) as an independent variable. When significant interaction effects were found, Bonferroni *post hoc* analyses were performed to elucidate the differences between groups within conditions. Given overall effect of drug treatment (AM251) in the absence of interactions between irradiation and AM251, unpaired two-tailed Student’s *t*-tests were performed for sham (0 Gy) and irradiated (9 Gy) cohorts separately. To evaluate the preferences for novelty, a three-way ANOVA following Sidak *post hoc* analyses was carried out using time spent exploring novel/familiar object × irradiation and AM251 treatment. Data are presented as the mean ± standard error of the mean (SEM). ^∗^*P* < 0.05; ^∗∗^*P* < 0.01; ^∗∗∗^*P* < 0.001; and ^****^P < 0.0001. Statistical significance was assigned at *P* ≤ 0.05.

## Results

### Treatment With AM251 Reverses Radiation-Induced Cognitive Dysfunction

#### NOR and OiP Task

**FIGURE 2:**
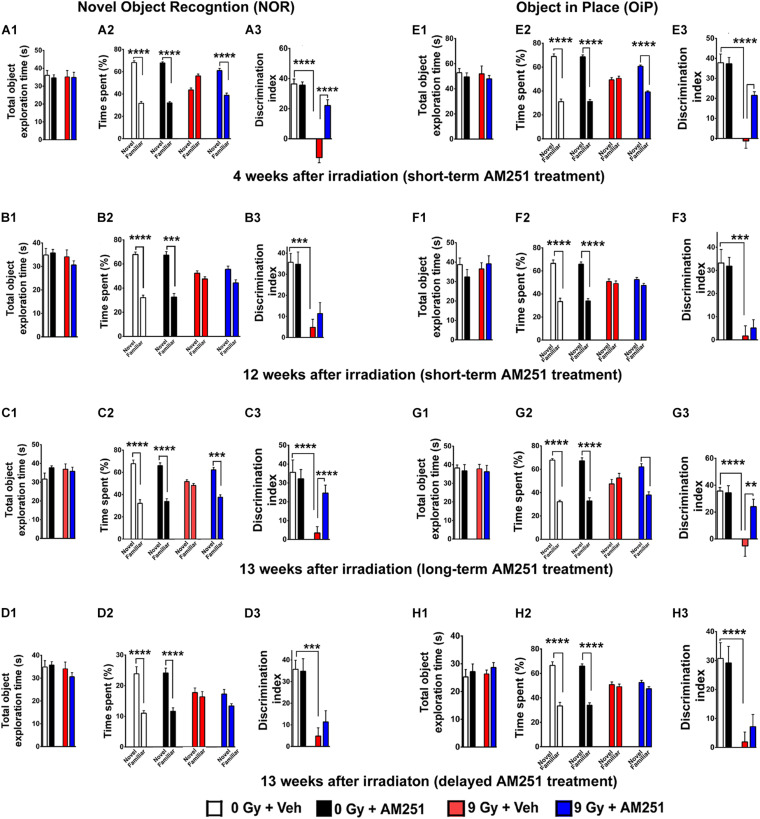
AM251 treatment reverses radiation-induced cognitive dysfunction. **NOR:** Total exploration time did not differ between all treatment groups **(A1–D1)**. At 4 weeks postirradiation, the preference for novelty was reduced significantly in irradiated mice, an effect that was ameliorated by short-term AM251 treatment **(A2)**, evidenced further by follow-up analysis of the DI **(A3)**. However, this same short-term AM251 treatment was unable to maintain efficacy 12 weeks following irradiation **(B2)**. While radiation-induced decrements persisted, the benefits of AM251 waned over this extended interval **(B3)**. Nonetheless, long-term AM251 treatments were found to maintain neurocognitive benefits **(C2)**, where radiation-induced decrements in the DI measured 13 weeks afterward were ameliorated significantly by AM251 **(C3)**. Delayed AM251 treatment was not effective in resolving radiation-induced cognitive deficits 13 weeks postirradiation **(D1–D3)**. **OiP:** Similarly, irradiation and/or AM251 treatment had no effect on total exploration time in the OiP task **(E1–H1)**. At 6 weeks following irradiation, the preference for novelty was reduced significantly in irradiated mice, which was ameliorated by short-term AM251 treatment **(E2)**, as indicated further by subsequent analysis of the DI **(E3)**. When this same short-term AM251 treatment was assessed 12 weeks later, radiation-induced deficits persisted while AM251 failed to show efficacy **(F2–F3)**. Long-term AM251 treatments were again found to restore neurocognitive benefits 13 weeks following irradiation **(G2)**, where radiation-induced deficits in the DI found 13 weeks later were ameliorated significantly by AM251 **(G3)**. Delayed AM251 treatment was not found to be effective in improving behavioral performance on the OiP task 13 weeks after irradiation **(H1–H3)**. Data presented as means ± SEM, *N* = 8–12. *P*-values for total exploration time **(A1–D1)** and DI **(E1–H1)** were derived from two-way ANOVA followed by Bonferroni correction for multiple comparisons. While three-way ANOVA following Sidak *post hoc* analyses was carried out using time spent exploring novel/familiar object × irradiation **(A2–D2;E2–H2)**. **P < 0.01; ***P < 0.001; and ****P < 0.0001.

A two-way ANOVA analyses on total time exploring both the objects revealed no significant interaction between irradiation and AM251 (4 weeks: *F*_1, 44_ = 0.04, *P* = 0.83; 12 weeks: *F*_1, 28_ = 0.89, *P* = 0.35; 13 weeks long-term: *F*_1, 44_ = 2.10, *P* = 0.15; and 13 weeks delayed: *F*_1, 44_ = 0.01, *P* = 0.92) as well no main effect of irradiation (4 weeks: *F*_1, 44_ = 0.01, *P* = 0.91; 12 weeks: *F*_1, 28_ = 1.60, *P* = 0.21; 13 weeks long-term: *F*_1, 44_ = 0.40; *P* = 0.50; and 13 weeks delayed: *F*_1, 44_ = 0.34, *P* = 0.56), and AM251 effect (*F*_1, 44_ = 1.01, *P* = 0.32; 12 weeks: *F*_1, 28_ = 0.32, *P* = 0.57; 13 weeks long-term: *F*_1, 44_ = 1.01, *P* = 0.32; 13 weeks delayed, Figures 2A1,B1,C1,D1). This indicated that exposure to irradiation and/or AM251 did not affect inherent exploration during NOR task.

A three-way ANOVA analyses on the time spent exploring familiar and novel objects at 4 weeks irradiation showed a triple interaction between novelty preference, irradiation, and AM251 (*F*_1, 88_ = 61.20, *P* < 0.0001), and significant interaction between both novelty preference and irradiation [*F*(1, 88) = 191.0, *P* < 0.0001], and novelty preference and AM251 treatment (*F*_1, 88_ = 55.81, *P* < 0.0001). Furthermore, Sidak *post hoc* analysis revealed that both unirradiated groups (0 Gy ± AM251) displayed a clear preference for novelty which is evident by the greater percentage of time spent exploring the novel object (*P* < 0.0001 for 0 Gy + Veh, *P* = 0.0001 for 0 Gy + AM251; Figure 2A2). While irradiated mice that received vehicle had no preference for novelty (*P* = 0.99, Figure 2A2), these data support the previous findings by us and others where cranial irradiation significantly impaired object recognition memory (Acharya et al., 2016; Tang et al., 2017). However, irradiated mice treated with AM251 did not exhibit such impairments and retained the capability to distinguish the novel object (*P* < 0.0001; Figure 2A2). For DI, two-way ANOVA revealed a significant interaction between irradiation and AM251 (*F*_1, 44_ = 30.60, *P* < 0.0001) and as well as significant radiation (*F*_1, 44_ = 95.54, *P* < 0.0001) and AM251 effect (*F*_1, 44_ = 27.91, *P* < 0.0001; Figure 2A3). The *post hoc* analysis showed irradiated mice treated with AM251 showed intact memory and displayed higher DI when compared with vehicle-treated irradiated group (9 Gy + Veh vs. 9 Gy + AM251, *P* < 0.0001, Figure 2A3). However, the beneficial effects of acute AM251 treatment waned and were not observed at 12 weeks postirradiation (Figures 2B1–B3). While acute AM251 treatments provided no benefits at 12 weeks postexposure, irradiation still caused significant and persistent reductions in the preference for novelty. A three-way ANOVA analysis on the time spent with novel and familiar objects showed no significant triple interaction between novelty preference, irradiation, and AM251 treatment (*F*_1, 56_ = 1.20, *P* = 0.28) but showed a significance between novelty and irradiation (*F*_1, 56_ = 63.86, *P* < 0.0001). Furthermore, Sidak *post hoc* analysis revealed that both unirradiated groups displayed a clear preference for novelty while irradiated mice showed no such preference (*P* < 0.0001 for 0 Gy + Veh, *P* = 0.0001 for 0 Gy + Veh, *P* = 0.50 for 9 Gy + Veh, *P* = 0.59 for 9 Gy + AM251; Figure 2B2). Two-way ANOVA analysis on DI (12 weeks) revealed no significant interaction between irradiation and AM251 (*F*_1, 28_ = 0.60, *P* = 0.44) and no main effect of AM251 treatment (*F*_1, 28_ = 0.34, *P* = 0.56) but did show a significant radiation effect (*F*_1, 28_ = 31.92, *P* < 0.0001; Figure 2B3).

Since the foregoing short-term treatment regimen forestalled the development of radiation-induced cognitive deficits at 4 but not 12 weeks postirradiation, we next sought to establish whether additional AM251 treatments could maintain the beneficial outcomes at more protracted postexposure time points. Data from these investigations indicated that more chronic treatment regimens resulted in longer-term benefits. A three-way ANOVA for time spent with novel and familiar object showed a triple interaction between novelty preference, irradiation, and AM251 (*F*_1, 88_ = 12.54, *P* = 0.0006) and significant interaction between both novelty preference and irradiation (*F*_1, 88_ = 32.93, *P* < 0.0001), and novelty preference and AM251 (*F*_1, 88_ = 6.54, *P* = 0.01). Sidak *post hoc* analysis revealed that both unirradiated groups (0 Gy ± AM251) displayed a clear preference for novelty while the irradiated mice (9 Gy + Veh) showed no such preference (*P* < 0.0001 for 0 Gy + Veh, *P* = 0.0001 for 0 Gy + AM251, *P* = 0.78 for 9 Gy + Veh; Figure 2C2). Moreover, the irradiated mice treated with AM251 did not exhibit such impairments and retained the capability to distinguish the novel object (*P* = 0.0001, Figure 2C2). For the DI, two-way ANOVA revealed a significant interaction between irradiation and AM251 treatment (*F*_1, 44_ = 7.42, *P* = 0.009) and as well as a significant irradiation (*F*_1, 44_ = 20.02, *P* = 0.0001) and AM251 effect (*F*_1, 44_ = 4.571, *P* = 0.04). *Post hoc* analysis revealed that long-term treatment with AM251 was efficacious at the resolution of memory function at the protracted postirradiation time of 12 weeks (9 Gy + Veh vs. 9 Gy + AM251, *P* = 0.008, Figure 2C3). In our final treatment arm, data indicated that delayed AM251 treatment 13 weeks postirradiation was not able to resolve radiation-induced cognitive deficits (Figures 2D2–D3). A three-way analysis on the time spent with and novel and familiar objects showed no significant triple interaction between the novelty preference, irradiation, and AM251 treatment (*F*_1, 88_ = 1.02, *P* = 0.30) but showed a significance between novelty and irradiation (*F*_1, 88_ = 56.22, *P* < 0.0001). Sidak *post hoc* analysis revealed that both unirradiated groups displayed a clear preference for novelty while irradiated mice showed no preference for novelty (*P* < 0.0001 for 0 Gy + Veh, *P* = 0.0001 for 0 Gy + Veh, *P* = 0.96, 9 Gy + Veh, *P* = 1.30 for 9 Gy + AM251; Figure 2D2). For the DI, two-way ANOVA revealed no significant interaction between irradiation and AM251 (*F*_1, 44_ = 0.51, *P* = 0.48) as well as no significant AM251 effect (*F*_1, 44_ = 0.15, *P* = 0.70) but did show a significant irradiation effect (*F*_1, 44_ = 28.12, *P* < 0.0001; Figure 2D3).

Following the NOR task, mice were habituated and tested on the OiP task, also known to be reliant on intact hippocampal and perirhinal cortex-dependent brain function. Two-way ANOVA analyses on total time exploring both the objects revealed no significant interaction between irradiation and AM251 (4 weeks: *F*_1, 44_ = 0.007, *P* = 0.93; 12 weeks: *F*_1, 28_ = 0.29, *P* = 0.60; 13 weeks long-term: *F*_1, 44_ = 0.0002, *P* = 1.0; and 13 weeks delayed: *F*_1, 44_ = 1.10, *P* = 0.30), as well as no main effect of irradiation (4 weeks: *F*_1, 44_ = 0.09, *P* = 0.76; 12 weeks: *F*_1, 28_ = 42.27, *P* = 0.14; 13 weeks long-term: *F*_1, 44_ = 0.03, *P* = 0.87; and 13 weeks delayed: *F*_1, 44_ = 3.32, *P* = 0.07), and AM251 effect (4 weeks: *F*_1, 44_ = 0.10, *P* = 0.75; 12 weeks: *F*_1, 28_ = 0.06, *P* = 0.81; 13 weeks long-term: *F*_1, 44_ = 0.30, *P* = 0.58; and 13 weeks delayed; *F*_1, 44_ = 0.45, *P* = 0.50; Figures 2E1,F1,G1,H1). This indicates that exposure to irradiation and/or AM251 did not impair inherent exploration during the OiP task.

A three-way ANOVA analyses on time spent exploring familiar and novel object locations showed a triple interaction between novelty preference, irradiation, and AM251 (*F*_1, 88_ = 12.54, *P* = 0.0006), and significant interaction between novelty preference and irradiation (*F*_1, 88_ = 32.93, *P* < 0.0001), and novelty preference and AM251 treatment (*F*_1, 88_ = 6.54, *P* = 0.01). Sidak *post hoc* analysis revealed that both unirradiated groups (0 Gy ± AM251) displayed a clear preference for objects that were moved to the novel location, evident by the greater percentage of time spent exploring new object locations, while irradiated vehicle-treated mice displayed no such preference. Importantly, irradiated mice treated with AM251 did not exhibit such impairments and retained the capability to distinguish to novelty (*P* < 0.0001 for 0 Gy + Veh, *P* = 0.0001 for 0 Gy + Veh, *P* = 0.79 for 9 Gy + Veh, *P* = 0.0001 for 9 Gy + AM25; Figure 2E2). For the DI, a two-way ANOVA revealed a significant radiation effect (*F*_1, 44_ = 65.96, *P* < 0.0001), AM251 effect (*F*_1, 44_ = 10.83, *P* = 0.002), and significant radiation × AM251 interaction (*F*_1, 44_ = 11.86, *P* = 0.001). *Post hoc* analysis revealed that AM251 significantly reduced the radiation-induced deficits, evident by the higher DI when compared with vehicle-treated irradiated mice (*P* < 0.0001, Figure 2E3). As found with the NOR task, short-term AM251 treatment waned, and did not prevent radiation-induced deficits in OIP at the extended 12-week postirradiation time (Figures 2F1–F3). A three-way ANOVA analysis on the time spent with the novel or familiar object locations showed no significant interaction between novelty preference, irradiation, and AM251 (*F*_1, 56_ = 0.56, *P* = 0.45), and no significant interaction between novelty and AM251 (*F*_1, 56_ = 0.11, *P* = 0.74) but did show a significant effect between novelty and irradiation (*F*_1, 56_ = 84.55, *P* < 0.0001). Cognitive deficits were nonetheless still apparent in the irradiated groups (9 Gy ± AM251) 12 weeks later, evidenced by the reduced preference for novelty (*P* < 0.0001 for 0 Gy + Veh, *P* = 0.0001 for 0 Gy + Veh, *P* = 0.97 for 9 Gy + Veh, *P* = 0.37 for 9 Gy + AM251, three-way ANOVA followed by Sidak *post hoc* analyses; Figure 2F2). Furthermore, two-way ANOVA analysis of the DI for the 12-week time revealed no significant interaction between irradiation and AM251 (*F*_1, 28_ = 0.28, *P* = 0.60) as well as no main effect of AM251 (*F*_1, 28_ = 0.05, *P* = 0.81) but did show a significant effect of the irradiation (*F*_1, 28_ = 42.27, *P* < 0.0001; Figure 2F3). Again, we found that the acute, short-term AM251 regimen prevented radiation-induced OiP memory deficits at early time points (4 weeks) but not at the later 13-week postirradiation time. Furthermore, three-way ANOVA analyses for time spent with objects at the novel and familiar locations showed a significant triple interaction between novelty preference, irradiation, and AM251 treatment (*F*_1, 88_ = 14.98, *P* = 0.0002), and significant interaction between novelty preference and irradiation (*F*_1, 88_ = 41.66, *P* < 0.0001), and novelty preference and AM251 treatment (*F*_1, 88_ = 12.45, *P* = 0.0007). Sidak *post hoc* analysis revealed that both unirradiated groups (0 Gy ± AM251) displayed a clear preference for objects that were moved to the novel location, evident by the greater percentage of time spent exploring the new object locations, while irradiated vehicle-treated mice showed no such preference.

As found before, irradiated mice subjected to long-term AM251 treatment did not exhibit such impairments and showed high preference to new object locations (*P* < 0.0001 for 0 Gy + Veh, *P* = 0.0001 for 0 Gy + Veh, *P* = 0.77 for 9 Gy + Veh, and *P* = 0.0001 for 9 Gy + AM251; Figure 2G2). For the DI, a two-way ANOVA revealed a significant interaction between irradiation and AM251 treatment (*F*_1, 44_ = 7.49, *P* = 0.009) as well as a significant irradiation (*F*_1, 44_ = 20.82, *P* < 0.0001) and AM251 effect (*F*_1, 44_ = 6.22, *P* = 0.02). *Post hoc* analysis revealed that AM251 resolved significantly the radiation-induced deficits in object recognition memory, evidenced by the higher DI when compared with vehicle-treated irradiated mice (*P* < 0.004; Figure 2G3). As found with NOR, delayed AM251 treatment at 13 weeks postirradiation was not found effective at ameliorating radiation-induced cognitive deficits on the OiP task. A three-way ANOVA analysis on the time spent with familiar or novel object locations showed no significant interaction between novelty preference, irradiation, and AM251 (*F*_1, 86_ = 0.003, *P* = 0.96), and no significant interaction between novelty and AM251 (*F*_1, 86_ = 0.28, *P* = 0.59) but did show a significant interaction between novelty and irradiation (*F*_1, 86_ = 45.68, *P* < 0.0001). *Post hoc* analysis revealed that AM251 was again unable to resolve radiation-induced deficits at 13 weeks postirradiation (*P* < 0.0001 for 0 Gy + Veh, *P* = 0.0001 for 0 Gy + Veh, *P* = 0.43 for 9 Gy + Veh, *P* = 0.82 for 9 Gy + AM251, Figure 2H2). Furthermore, two-way of DI for the delayed AM251 treatment at 13 weeks revealed no significant interaction between irradiation and AM251 (*F*_1, 44_ = 0.009, *P* = 0.92) as well as no main effect of AM251 (*F*_1, 44_ = 0.109, *P* = 0.74) but did show a significant effect of the irradiation (*F*_1, 44_ = 24.37, *P* < 0.0001; Figure 2H3). *Post hoc* analyses revealed that radiation-induced cognitive deficits persisted at 13 weeks postirradiation (*P* = 0.0081), and that AM251 was unable to resolve those impairments (*P* > 0.99, Figure 2H3).

### AM251 Reduces Anxiety and Depressive-Like Behavior in Irradiated Mice

**FIGURE 3:**
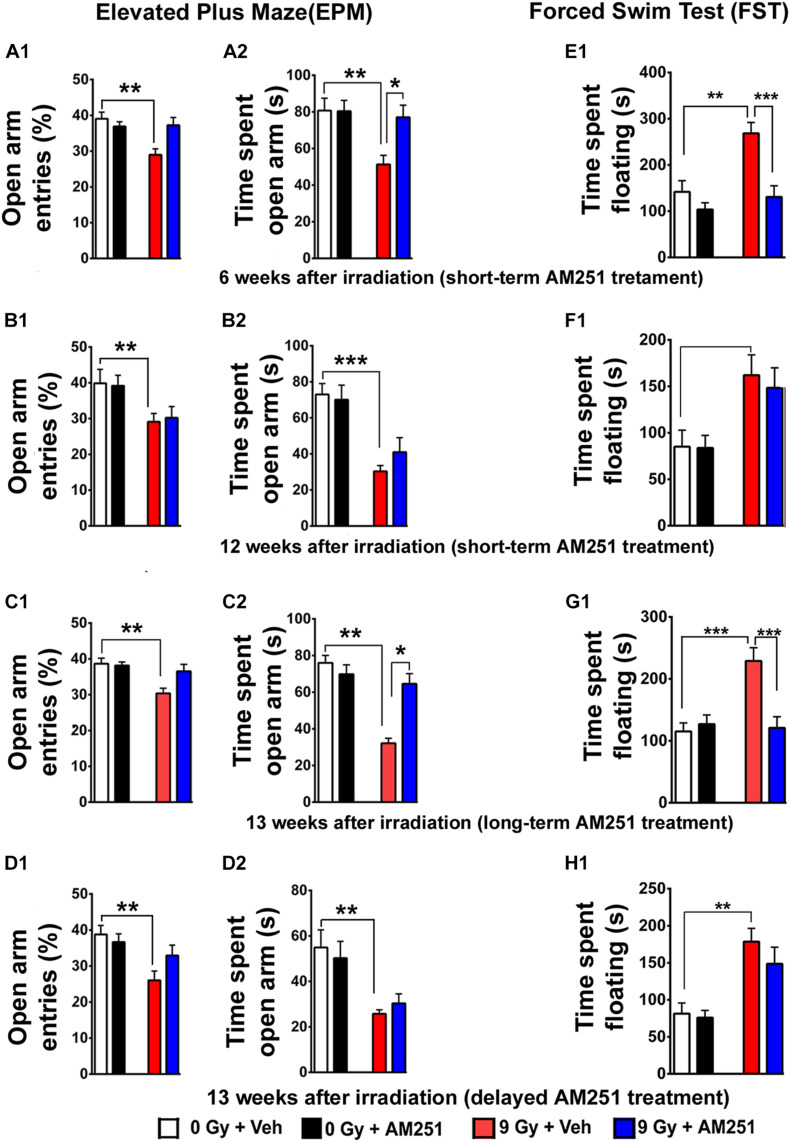
AM251 ameliorates anxiety and depressive-like behavior in irradiated mice. **EPM:** Short-term AM251 treatment reduced anxiety-like-behavior in mice 6 weeks following irradiation evidenced by the significant increased time spent in open arms **(A1,A2)**. However, benefits derived from this same short-term treatment did not persist 12 weeks after irradiation **(B1,B2)**. At similar protracted postirradiation times, long-term AM251 treatments were found to significantly reduce anxiety-like behavior **(C1,C2)**. Delayed AM251 treatment was not effective at resolving radiation-induced anxiety-like behavior 13 weeks after irradiation **(D1,D2)**. **FST:** Short-term AM251 treatment reduced significantly depressive-like behavior in irradiated mice at 6 weeks after irradiation **(E1)** but not at 12 weeks postirradiation **(F1)**. Long-term treatments with AM251 improved were found to reduce depressive-like behavior 13 weeks following irradiation **(G1)** but not when a single treatment was delayed **(H1)**. Data presented as means ± SEM, *N* = 8–12. *P*-values derived from two-way ANOVA followed by Bonferroni correction for multiple comparisons. *P < 0.05; **P < 0.01; and ***P < 0.001.

Next, we applied the EPM test, a standard for measuring anxiety in mouse models. For the short-term AM251 treatment arm, two-way ANOVA analysis on the percentage of open-arm entries revealed a significant interaction between irradiation and AM251 (*F*_1, 44_ = 8.05, *P* = 0.007) as well as a significant radiation effect (*F*_1, 44_ = 7.06, *P* = 0.01) but no AM251 effect (*F*_1, 44_ = 2.81, *P* = 0.10; Figure 3A1). Furthermore, comparison of the total time spent in the open arm revealed a significant main effect of irradiation (*F*_1, 44_ = 7.22, *P* = 0.01) and AM251 (*F*_1, 44_ = 4.35, *P* = 0.04) as well as a significant interaction of irradiation and AM251 (*F*_1, 44_ = 4.58, *P* = 0.04; Figure 3A2). *Post hoc* analyses revealed that irradiated mice treated with AM251 spent a significantly longer time (*P* = 0.03) in the open arms when compared with vehicle−treated irradiated mice (Figure 3A2). However, acute, short-term treatment with AM251 did not reduce radiation-induced anxiety-like behavior at 12 weeks following irradiation (Figure 3B). Two-way ANOVA analysis on the percentage of entries to open arm revealed no significant interaction between irradiation and AM251 (*F*_1, 28_ = 1, 77, *P* = 0.19) as well as a no AM251 effect (*F*_1, 28_ = 1.10, *P* = 0.30) but did show a significant radiation effect (*F*_1, 28_ = 16.76, *P* = 0.003; Figure 3B1). Further comparison of the total time spent in the open arm revealed no significant interaction between irradiation and AM251 (*F*_1, 28_ = 1.03, *P* = 0.31) and no main effect of AM251 (*F*_1, 28_ = 0.34, *P* = 0.56) but did show a significant radiation effect (*F*_1, 28_ = 29.21, *P* = 0.0001; Figure 3B2). To gage the impact of the chronic, longer-term AM251 treatment on the EPM, additional cohorts were tested 13 weeks following irradiation. Two-way ANOVA analysis on the percentage of entries to the open arms revealed a significant interaction between irradiation and AM251 (*F*_1, 44_ = 4.13, *P* = 0.04) as well as a significant radiation effect (*F*_1, 44_ = 7.47, *P* = 0.009) but no AM251 effect (*F*_1, 44_ = 2.16, *P* = 0.14; Figure 3C1). Furthermore, comparison of the total time spent in open arm revealed a significant main effect of irradiation (*F*_1, 44_ = 7.22, *P* = 0.01) and AM251 (*F*_1, 44_ = 4.35, *P* = 0.04) as well as a significant interaction of irradiation and AM251 (*F*_1, 44_ = 4.58, *P* = 0.04; Figure 3C2). *Post hoc* analyses showed that irradiated mice treated with AM251 spent a significantly longer time (*P* = 0.03) in the open arm when compared with vehicle−treated irradiated mice (Figure 3C2). Additional studies aimed at evaluating the impact of the delayed AM251 treatment at 13 weeks postirradiation showed this approach to be ineffective at resolving radiation-induced anxiety-like behavior. Two-way ANOVA analysis on the percentage of entries to the open arms revealed no significant interaction between irradiation and AM251 (*F*_1, 44_ = 3.12, *P* = 0.08) and no significant effect of AM251 (*F*_1, 44_ = 0.87, *P* = 0.35) but did show a significant irradiation effect (*F*_1, 44_ = 10.34, *P* = 0.002; Figure 3D1). Further comparison of the total time spent in the open arm revealed no significant interaction between irradiation and AM251 (*F*_1, 44_ = 0.63, *P* = 0.43), no main effect of AM251 (*F*1, 44 = 0.00005, *P* = 0.99), but did show a significant effect of irradiation (*F*_1, 44_ = 17.89, *P* = 0.0001; Figure 3D2).

Analyses of immobility (or floating) time on the FST were used as a measure of depressive-like behavior for each of the cohorts (Figures 3E–H). For the acute, short-term treatment arm, two-way ANOVA analysis of the time spent floating revealed a significant interaction between AM251 and irradiation (*F*_1, 44_ = 5.15, *P* = 0.03) and a significant irradiation (*F*_1, 44_ = 12.24, *P* = 0.001) and AM251 effect (*F*_1, 44_ = 16.05, *P* = 0.0002). *Post hoc* analysis revealed that for irradiated mice treated with AM251, the duration of immobility was significantly less (*P* < 0.0004; Figure 3E1). This reduction in AM251-mediated depressive-like behavior was not apparent in irradiated mice 12 weeks later (Figure 3F1). Two-way ANOVA analysis on time spent floating at this protracted time revealed no significant interaction between AM251 and irradiation (*F*_1, 28_ = 0.10, *P* = 0.74) and no main effect of AM251 (*F*_1_,_28_ = 0.15, *P* = 0.69) but did show a significant irradiation effect (*F*_1, 28_ = 13.97, *P* = 0.0008). For the chronic, longer-term AM251 treatment arm, data found this approach to be effective in reversing depressive-like behavior at 13 weeks postirradiation (Figure 3G1). Two-way ANOVA analysis on the time spent in floating revealed a significant interaction between AM251 and irradiation (*F*_1, 44_ = 12.05, *P* = 0.001) and a significant main effect of irradiation (*F*_1, 44_ = 9.73, *P* = 0.003) and AM251 (*F*_1, 44_ = 7.76, *P* = 0.008). Nonetheless, each irradiated cohort exhibited depressive-like behavior at 13 weeks postirradiation in AM251 reverses depressive-like-behavior in irradiated mice (*P* = 0.0002 for 0 Gy + Veh vs. 9 Gy + Veh; *P* = 0.0004 for 9 Gy + Veh vs. 9 Gy + AM251; Figure 3G1). As in all our previous behavioral tasks, delayed AM251 treatment was not found effective at resolving depressive-like behavior 13 weeks following irradiation. Two-way ANOVA analysis revealed no significant interaction between irradiation and AM251 (*F*_1, 44_ = 0.53, *P* = 0.47), no AM251 effect (*F*_1, 44_ = 1.11, *P* = 0.30), but did show a significant effect of irradiation (*F*_1, 44_ = 25.79, *P* < 0.0001; Figure 3H1).

### AM251 Treatment Preserves the Number of Newly Born DCX Positive Neurons and Promotes Cell Proliferation and Survival in the Hippocampus of Irradiated Mice

**FIGURE 4:**
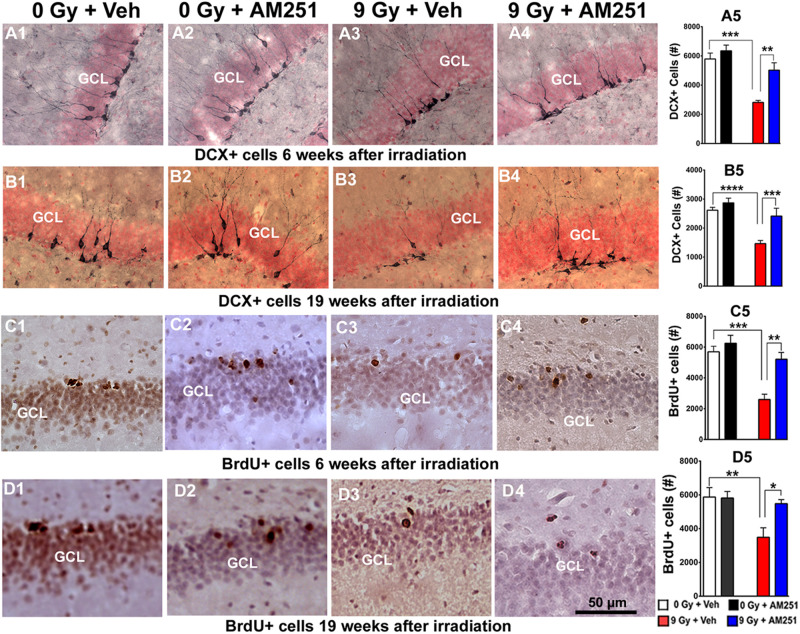
AM251 preserves the number of immature neurons and cell proliferation in the irradiated hippocampus of mice. **DCX:** Representative images showing density of immature neurons (DCX + cells, brown staining) in the SGZ-GCL of the hippocampus (pink, nuclear staining) at 6 **(A1–A4)** and 19 weeks **(B1–B4)** following irradiation. At 6- and 19-weeks postirradiation, significant radiation-induced reductions in the number DCX + immature neurons were found, an effect that was ameliorated significantly by short- **(A5)** and long-term **(B5)** AM251 treatments, respectively. **BrdU:** Representative confocal images showing BrdU + cells (darkly staining) in the SGZ-GCL of the hippocampus at 6 **(C1–C4)** and 15 weeks **(D1–D4)** following irradiation. At 6 and 19 weeks postirradiation, significant radiation-induced reductions in the number of BrdU + cells were found which was prevented by short- **(C5)** and long-term **(D5)** AM251 treatments, respectively. Data presented as means ± SEM, *N* = 6. *P*-values derived from two-way ANOVA followed by Bonferroni correction for multiple comparisons. *P < 0.05; **P < 0.01; ***P < 0.001; and ****P < 0.0001.

To assess the impact of acute (Figures 4A,C) or chronic (Figures 4B,D) AM251 treatments on DCX + neurons, brightfield staining was conducted at 6 or 19 weeks after cranial irradiation. Images show qualitatively how the yields varied across the treatment groups (Figures 4A1–A4,B1–B4). Data quantified from these cohorts showed that radiation-induced reductions in DCX + neurons was ameliorated significantly by short- (Figure 4A5) and long-term (Figure 4B5) AM251 treatments. At 6 weeks postirradiation, two-way ANOVA analysis on the number of DCX + neurons revealed a significant effect of irradiation (*F*_1, 20_ = 31.30, *P* < 0.0001) and AM251 (*F*_1, 20_ = 12.97, *P* = 0.002), as well as a significant interaction between irradiation and AM251 (*F*_1, 20_ = 4.58, *P* = 0.04). *Post hoc* analysis further confirmed that DCX + neurons in the SGZ-GCL were significantly reduced in irradiated mice when compared with unirradiated controls (*P* < 0.0001), and that treatment with AM251 prevented the radiation-induced reduction in the number of DCX + neurons (*P* = 0.004; Figure 4A5). Similarly, two-way ANOVA analysis at latter 19 weeks postirradiation time revealed a significant interaction between irradiation and AM251 (*F*_1, 20_ = 5.23, *P* = 0.03) as well as significant effect of irradiation (*F*_1, 20_ = 81.86, *P* = 0.0001) and AM251 (*F*_1, 20_ = 24.87, *P* = 0.0001). *Post hoc* analysis confirmed that irradiation reduced significantly the number of DCX + cell in SGZ-GCL (*P* < 0.0001), and that long-term treatment with AM251 significantly prevented this drop in the number of DCX + neurons (*P* = 0.004; Figure 4B5). Data indicates that both short- and long-term AM251 treatments afforded significant protection to immature neurons (∼1.7-fold increase) in the irradiated hippocampus.

Related imaging obtained 6 and 19 weeks postirradiation revealed similar qualitative patterns in the yields of BrdU + cells across the treatment groups (Figures 4C1–C4,D1–D4). Two-way ANOVA analysis on the number of BrdU + cells 6 weeks following irradiation revealed a significant interaction between irradiation and AM251 (*F*_1, 20_ = 5.95, *P* = 0.02) as well as significant main effect of irradiation (*F*_1, 20_ = 23.99, *P* = 0.0001) and AM251 (*F*_1, 20_ = 14.09, *P* = 0.001). *Post hoc* analysis confirmed that the yield of BrdU + cells in the SGZ-GCL was reduced significantly in irradiated mice when compared with unirradiated controls (*P* = 0.0003), and that treatment with AM251 prevented this effect (*P* = 0.002; Figure 4C5). Two-way ANOVA analysis at the longer time point (19 weeks) revealed a significant irradiation (*F*_1_, 20 = 8.82, *P* = 0.008) and AM251 effect (*F*_1_, 20 = 4.46, *P* = 0.04) and a significant AM251 and radiation interaction (*F*_1_, 20 = 5.01, *P* = 0.04). *Post hoc* analysis showed a significant reduction in the number of BrdU + cells in the SGZ-GCL of irradiated mice (*P* = 0.008), an effect that long-term treatment with AM251 was again able to prevent (*P* = 0.03; Figure 4D5). These results corroborated the foregoing data and showed that after either short- or long-term AM251 treatment, a 2- and 1.5-fold increase in cell proliferation was found in the irradiated hippocampus, respectively.

### AM251 Treatment Protects Neurogenesis and Prevents the Loss of Mature Neurons in the GCL of the Irradiated Hippocampus

**FIGURE 5:**
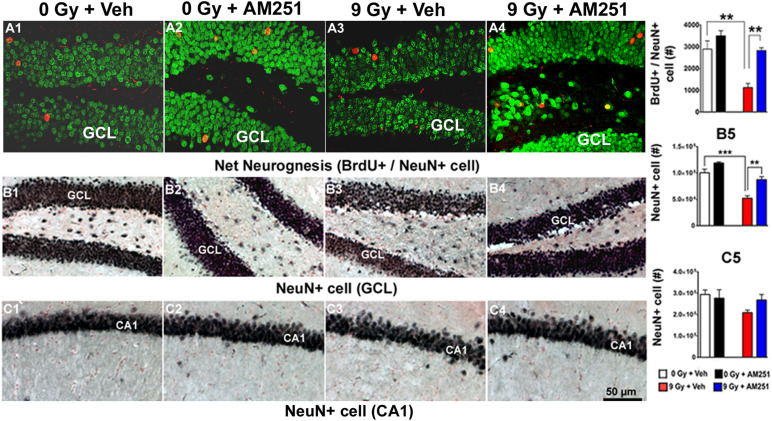
AM251 protects hippocampal neurogenesis and preserves the number of mature neuron (NeuN +) in the irradiated hippocampus of mice: **Neurogenesis:** Representative confocal images showing BrdU + cells (red staining) with NeuN (green staining) coexpression **(A1–A4)**. At 19 weeks postirradiation, significant radiation-induced reductions in the number BrdU + /NeuN + neurons were observed, an effect that was ameliorated by long-term AM251 treatments **(A5)**. **Stereology:** Brightfield images show the distribution of NeuN + neurons (black staining) in the GCL **(B1–B4)** and CA1 **(C1–C4)** subfields of the hippocampus among treatment groups. Quantification at 19 weeks postirradiation demonstrated a significant loss of NeuN + cells in the GCL of irradiated animals, an effect that was ameliorated in mice that received long-term AM251 treatments **(B5)**. The number of NeuN + cells in the CA1 was not found to differ significantly among the various treatment groups **(C5)**. Data are expressed as the mean ± SEM, *N* = 4. *P*-values for BrdU + /NeuN derived from two-way ANOVA followed by Bonferroni correction for multiple comparisons. Whereas, *t*-test was used to analyzed mature neurons in the GCL (B5). **P < 0.001; ***P < 0.001.

To determine the impact of chronic AM251 treatments on neurogenesis, the number of BrdU + cells that coexpressed NeuN was scored 19 weeks postirradiation from representative immunohistochemical images obtained across each treatment group (Figures 5A1–A4). Two-way ANOVA run on the absolute number of mature neurons (BrdU + /NeuN + cells) revealed a significant interaction between irradiation and AM251 (*F*_1, 12_ = 4.84, *P* = 0.04), as well as significant main effect of irradiation (*F*_1, 16_ = 7.10, *P* = 0.0004) and AM251 (*F*_1, 12_ = 21.80, *P* = 0.0005). *Post hoc* analysis showed a significant reduction in the absolute number of mature neurons in the SGZ-GCL of irradiated mice (*P* = 0.002), and that long-term treatment of AM251 enhanced neurogenesis significantly (2.5-fold) when compared with irradiated cohorts treated with vehicle (*P* = 0.002; Figure 5A5). Data corroborates prior results and shows that long-term AM251 treatment protects neurogenesis after irradiation.

To further assess the impact of chronic AM251 treatments on more mature neuronal populations, NeuN-immunostaining was conducted 19 weeks following irradiation, which revealed normal hippocampal cytoarchitecture across the treatment groups (Figures 5B1–B4, GCL; Figures 5C1–C4,CA1). The partial loss of neurons in the GCL of animals exposed to irradiation and this drop in NeuN + cell was prevented in AM251 treatment. Two-way ANOVA analysis revealed a significant effect of radiation (*F*_1, 12_ = 59.72, *P* = 0.0001) and AM251 (*F*_1, 12_ = 26.92, *P* = 0.002) but no significant interaction between irradiation and AM251 (*F*_1, 12_ = 2.58, *P* = 0.13). As the interaction between irradiation and AM251 did not reach significance, *post hoc* analyses were not performed. However, independent *t*-tests revealed a significant loss of mature neurons in the GCL of irradiated mice and that long-term treatment with AM251 prevented this loss (*P* = 0.0002 for 0 Gy + Veh vs. 9 Gy + Veh, *P* = 0.003 for 9 Gy + Veh vs. 9 Gy + AM251) and (*P* = 0.003, Figure 5B5). Similar analyses conducted on the number of NeuN + cells in the CA1 between the cohorts revealed no significant effect of radiation (*F*_1, 12_ = 3.15, *P* < 0.10) or AM251 (*F*_1, 20_ = 0.63, *P* = 0.44) and no significant interaction between irradiation and AM251 (*F*_1, 12_ = 0.63, *P* = 0.44; Figure 5C5).

### AM251 Suppresses the Expression of HMGB1 in the Hippocampus of Irradiated Mice

**FIGURE 6:**
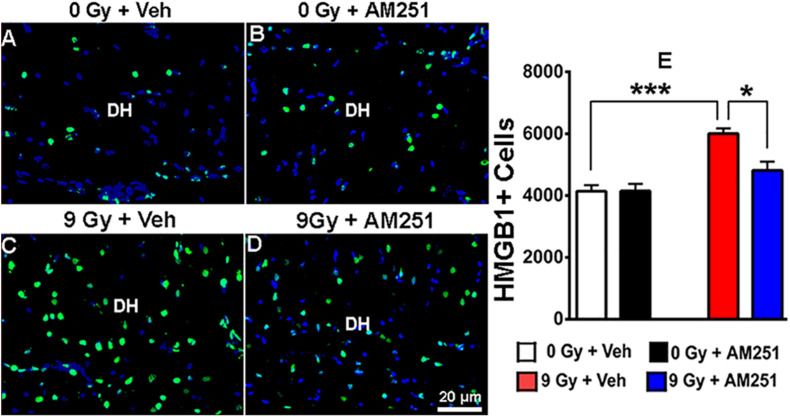
AM251 treatment reduces the expression of HMGB1 in irradiated mice. Representative confocal images of HMGB1 + cells (green staining) in the dentate hilus (DH) of the hippocampus (blue nuclear staining, **(A–D)**. Quantification at 19 weeks postirradiation demonstrated a significant increase in the number of HMGB1 + cells in irradiated animals, which was attenuated in mice that received long-term AM251 treatments **(E)**. Data are expressed as the mean ± SEM, *N* = 4. *P*-values derived from two-way ANOVA followed by Bonferroni correction for multiple comparisons. *P < 0.05; ***P < 0.001.

To ascertain whether chronic AM251 treatments might modulate proinflammatory signaling, immunostained tissues were analyzed for HMGB1 expression 19 weeks following irradiation. Images obtained across the cohorts revealed that while AM251 had little impact on HMGB1 levels in unirradiated controls, expression of this proinflammatory mediator following irradiation could be suppressed by long-term AM251 treatment (Figures 6A–D). Two-way ANOVA analysis revealed a significant effect of radiation (*F*_1, 12_ = 32.02, *P* = 0.0001) and AM251 (*F*_1, 12_ = 7.02, *P* = 0.02), as well as a significant interaction between irradiation and AM251 (*F*_1, 12_ = 7.17, *P* = 0.02). *Post hoc* analyses revealed enhanced expression of HMGB1 in irradiated mice (*P* = 0.0004) which was significantly reduced by AM251 (*P* = 0.016, Figure 6E). These data suggest that one of the potential underlying benefits of extended AM251 treatment involves the capability to suppress a key initiator of neuroinflammation.

## Discussion

This study provides novel evidence that AM251 provides wide-ranging benefits to the irradiated CNS. Improvements in behavioral indices of mood, learning and memory, enhanced neurogenesis, and reductions in chronic inflammation highlight the potential promise of AM251 treatment in cranially irradiated mice. Data derived from the NOR and OiP tasks indicated that irradiation disrupts hippocampal, medial prefrontal cortex, and perirhinal cortex circuitry, which impacts spatial and associative recognition memory function (Rola et al., 2004; Acharya et al., 2009; Parihar et al., 2014). In contrast to those animals receiving irradiation only, the performance of animals receiving irradiation and AM251 was indistinguishable from vehicle controls, where both controls and AM251-treated cohorts showed a significant preference for exploring the novel location and/or object following short- (4 weeks) and long-term (12 weeks) treatment regimes. These same treatment paradigms were similarly found to elicit significant improvements on the EPM and FST task, able to ameliorate radiation-induced deficits in anxiety and depression-like behaviors, respectively, when assessed 4–12 weeks later.

Overall, irradiated cohorts routinely showed learning and memory deficits in the NOR and OIP and mood disruptions in the EPM and FST tasks, which coincided with reductions in cell proliferation and cell survival in the hippocampal dentate gyrus. Most of these adverse effects could be restored to control levels when AM251 was administered following specific postirradiation treatments. The neurological benefits afforded by select AM251 treatment paradigms were found to be temporally coincidental with significant increases in hippocampal proliferation, newly born neuron production, neurogenesis, sparing of mature GCL neurons, and reduced expression of HMGB1 in irradiated mice.

The foregoing findings were, however, dependent on the specifics of each AM251 treatment paradigm. Acute benefits derived from four daily AM251 injections directly after irradiation observed at 4 weeks, did not persist when assessed 12 weeks after irradiation. These data point the progressive and relatively irreversible adverse effects of cranial irradiation on neurocognitive function, where metabolic turnover of the AM251 ligand eventually diminished the beneficial blockade on endocannabinoid signaling, releasing the brain to succumb to prior radiation injury. These results prompted follow-up studies, investigating the possibility of whether four monthly injections (4/day) could maintain the beneficial effects of AM251 at more protracted postirradiation intervals. Data derived from all behavioral tasks conclusively showed that such an approach was indeed beneficial when assessed 12 weeks postexposure, indicating that repeated AM251 treatments were sufficient to suppress persistent damage-induced signaling in the irradiated brain. Furthermore, to directly assess the reversibility of radiation-induced brain injury as well as the efficacy of other treatment approaches, AM251 was administered just prior to behavioral assessment conducted 12 weeks postexposure. Data showed consistently that such delayed treatments were ineffective at providing any benefits. These results suggest a limited therapeutic window following cranial irradiation, and that interventions initiated past this timeframe (12 weeks in this instance) are either unable to circumvent pre-existing damage to neural circuitry mediating behavior, or that the AM251 dosing regimen was suboptimal to provide measurable efficacy. Noteworthy also is that many of the prior studies addressing the impact of AM251 on anxiogenic behavior (Rodgers et al., 2005; Sink et al., 2010) were performed acutely, where anxiogenic behavior was tested soon after administration of AM251, distinctly different circumstances than those investigated in the present study. Here, the effects of AM251 appear to preferentially rescue “pathologic” as opposed to “control conditions” which in our case represents the irradiated vs. normal brain.

To the best of our knowledge, this has been the first study investigating the role of AM251 in resolving radiation-induced neurological complications. To evaluate the potential causes of these beneficial effects, we quantified the impact of AM251 on a variety of physiological factors affecting hippocampal function. Past results from us and others have conclusively determined that irradiation leads to a marked drop in newly born neurons (Nacher et al., 2001; Monje et al., 2007; Acharya et al., 2009; Parihar et al., 2014). Here, we found that improvements in neurocognitive function mediated by AM251 were associated with an increased yield of DCX + neurons (∼1.7-fold) and BrdU + (∼1.8-fold) cells in the irradiated hippocampus. Consistent with these trends, we found that irradiated mice subjected to various AM251 treatments exhibited significant improvements in neurogenesis (2.5-fold), again coincident with enhanced behavioral performance. Importantly, the benefits of AM251 treatments were not limited to the relatively minor proportion of newly added hippocampal cells, but rather, extended to the greater proportion of pre-existing mature hippocampal neurons. In the present study, we investigated the protective effects of AM251 on select cognitive, cellular, and molecular endpoints impacted by cranial irradiation. Furthermore, we determined that AM251 prevented the loss of mature neurons in GCL but not in the CA1 subregion of the hippocampus. The enhanced radiation-induced reductions in the number of GCL cells vs. the CA1 may be the result of diminished cell proliferation in the GCL, as opposed to more direct effects of radiation on mature neurons in either region (NeuN+). The presence of rapidly dividing cells in the GCL, which are known to be particularly vulnerable to radiation-induced depletion, distinguishes this region from the postmitotic population of cells in the CA1. Furthermore, our data confirms that radiation-induced depletion of newly born cells in the GCL persists 19 weeks and likely accounts for the overall reduction in the net number of GCL cells reported. Collectively, data supports the neuroprotective properties of AM251 and corroborates previous studies finding that AM251 enhances hippocampal neurogenesis (Hill et al., 2010) and the survival of mature neurons in various models of neuronal damage and neurodegenerative diseases (Bialuk and Winnicka, 2011; Hutch and Hegg, 2016). Interestingly, AM251 was not found to alter cognition or hippocampal turnover in unirradiated mice, despite prior reports demonstrating that AM251 might differentially regulate cell proliferation and survival, particularly type-2b (late progenitor cells) vs. type-3 (immature neurons; Hill et al., 2010; Wolf et al., 2010; Hutch and Hegg, 2016).

Notwithstanding the proneurogenic effects of AM251, its capability to dampen proinflammatory signaling is likely to play a beneficial role in the irradiated brain. This is not surprising, as the link between reduced inflammation and improved neurogenesis has been established (Monje et al., 2003; Kohman and Rhodes, 2013; Liao et al., 2013). In this regard, accumulating evidence from a broad range of studies supports the role of HMGB1 in promoting the expression of proinflammatory cytokines (Paudel et al., 2018; Tao et al., 2019) and that inhibition of HMGB1 can protect the brain against acute and chronic oxidative stress by modulating the activity of proinflammatory signaling pathways (Kim et al., 2012). In our present study, reduced expression of HMGB1 points to the anti-inflammatory effects of AM251 and are consistent with earlier findings showing that AM251 can reduce proinflammatory cytokines in rat plasma and brain tissue (Mehrpouya-Bahrami et al., 2017; Miranda et al., 2019; Ruz-Maldonado et al., 2020). Thus, the capability of AM251 to inhibit HMGB1 expression suggests that a certain fraction of the neurological benefits obtained after cranial irradiation is derived by suppressing the HMGB1/TLR4 neuroinflammatory axis.

## Conclusion

Our results add to a growing body of evidence suggesting that the adverse impact of cranial irradiation on multiple neurocognitive indices results in part, through the inhibition of neurogenesis and a persistent neuroinflammation (Monje and Palmer, 2003; Monje et al., 2007; Greene-Schloesser et al., 2012, 2013; Acharya et al., 2016; Hinkle et al., 2019). The capability of AM251 to improve both neurogenesis and reduce inflammation points to its mechanism of action in resolving radiation-induced cognitive dysfunction. Noteworthy also from these studies was the elucidation of optimal AM251 administration regimens, where short-term benefits waned after 4 weeks, requiring multiple treatments to maintain efficacy over a 12-week timeframe. These data not only pointed to the persistence of radiation-induced perturbations to the CNS but also found them relatively irreversible, as delayed treatment with AM251 past an apparent therapeutic window (i.e., 12 weeks) failed to provide any measurable efficacy in forestalling radiation-induced neurological complications. On the basis of these results, we speculate that the judicious use of AM251 or more recently developed analogs possessing less side effects and/or slower turnover rates may one day provide clinicians with a powerful interventional strategy for curtailing the unwanted normal tissue toxicities associated with the radiotherapeutic management of brain malignancies.

## Data Availability Statement

The raw data supporting the conclusions of this article will be made available by the authors, without undue reservation.

## Ethics Statement

The animal studies were reviewed and approved by University of California Irvine Institutional Animal Care and Use Committee.

## Author Contributions

VP: conceptualization, investigation, methodology, data curation, formal analysis, funding acquisition, writing—original draft, and writing—review. AS, LF, AL, RA, and JS: investigation. BA: formal analysis, validation, and investigation. MA: formal analysis, investigation, and methodology. SS: investigation and methodology. EG: validation, methodology, and investigation. CL: conceptualization, resources, data curation, and analysis, supervision, funding acquisition, writing—original draft, writing—review, and editing. All authors contributed to the article and approved the submitted version.

## Conflict of Interest

The authors declare that the research was conducted in the absence of any commercial or financial relationships that could be construed as a potential conflict of interest.
