# Supplementary material for: The Cannabinoid Receptor 1 Reverse Agonist AM251 Ameliorates Radiation-Induced Cognitive Decrements
Source: Front Cell Neurosci. 2021 Jun 28;15:668286. doi: 10.3389/fncel.2021.668286 (PMC8273551; doi:10.3389/fncel.2021.668286)
Supplement: Supplementary file 1 [file Data_Sheet_1.pdf]

The cannabinoid receptor 1 reverse agonist AM251 ameliorates radiation-induced cognitive decrements

Vipan K. Parihar*, Amber Syage, Lidia Flores, Angelica Lilagan, Barrett D. Allen, Maria C. Angulo, Sarah M. Smith, Rebecca J. Arechavala, Erich Giedzinski and Charles L. Limoli*

Department of Radiation Oncology, University of California, Irvine, CA 92697-2695, USA

*__Corresponding authors:__

***Corresponding authors:**
Dr. Vipan K. Parihar
Department of Radiation Oncology
University of California, Irvine
Irvine CA 92697-2695, USA. Phone: (949) 824-7395
E-mail: vipank@uci.edu

Prof. Charles L. Limoli
Department of Radiation Oncology
University of California, Irvine
Irvine CA 92697-2695, USA.
Phone: (949) 824-3053
E-mail: climoli@uci.edu

Running title: AM251 reverses memory impairments in irradiated mice
Key words: Cranial irradiation, Mood and memory deficits, AM251, Neurogenesis, HMGB1.
Number of Figures: 6
Number of pages: 30 (single spaced)

Methods:
All animal procedures described in this study were in accordance with NIH guidelines and approved by the University of California Irvine and Stanford University Institutional Animal Care and Use Committees. Unique cohorts used for the 4, 12 and 13-week studies were divided into four experimental groups (0 Gy± AM251 or 9Gy ±AM251). Each cohort consisted of 6-month-old wild-type male mice (C57BL/6J, JAX), housed in groups of 2–4 mice per individually-ventilated cage and maintained in standard housing conditions (20ºC ± 1ºC ; 70% ± 10% humidity; 12:12 h light-dark schedule) and provided ad libitum access to food (Envigo Teklad 2020x, Indianapolis, IN) and water(1,2)

Irradiation: For cranial irradiation, mice were anesthetized (5% induction and 2% maintenance isoflurane, vol/vol), placed ventrally on the treatment table (XRAD 320 irradiator, Precision X-ray, North Branford, CT) without restraint, and positioned under a collimated (1.0 cm^2 diameter) beam for head-only irradiation such that cerebellum, eyes and rest of the body were shielded from the radiation exposure. The irradiator was equipped with a hardening filter (0.75mm Sn + 0.25mm Cu + 1.5mm Al; HVL = 3.7mm Cu, half value layer) to eliminate low energy X-rays to minimize skin damage. X-irradiation was delivered at a dose rate of 1.10 Gy/min (1,3,4).

Assessment of newly born doublecortin (DCX+) neurons and hippocampal neurogenesis: For analyses of early hippocampal neurogenesis (6week after irradiation, **Fig. 1A**), 6 mice/group received 5-bromodeoxyuridine (BrdU; 50mg/kg, once every 6 h for 18 h, i.p.). Animals were perfused 6 h after the last BrdU injection and brain tissues processed for quantification of cell proliferation. For the analysis of latter neurogenesis (**Fig. 1B**), two days after the completion of behavioral tests (15 weeks after irradiation), animals received daily injections of BrdU for 7 days (50 mg/kg, i.p.). Animals were perfused a month after the last BrdU injection (i.e 19 weeks after irradiation), brain tissues were again processed for the quantification of neurogenesis in addition to neuroinflammation as described previously. Another series (every 15th) of sections from each animal were processed for DCX immunostaining using the ABC method, as detailed in our earlier study (5-8).

Behavioral testing: NOR and OiP tasks interrogate functional connectivity between different regions of the brain including the medial prefrontal cortex (mPFC), perirhinal cortex (PRC) and hippocampus. NOR is a measure of preference for novelty, which relies on intact mPFC function, while OiP is a test of associative recognition memory that depends on interactions between the hippocampus, mPFC and PRC functions (9,10). Behavior tests were conducted as described previously (1,9-14). All the behavior tests were conducted in a dimly lit (rooms illumination 915 Lux.), dedicated room separated from housing. Four arena boxes made of acrylic (30 x 30 x 30 cm) were placed two by two, layered with fresh, autoclaved, corncob bedding, and placed on the floor. Care was taken to keep the arenas in exactly the same location, and mice were never rotated to other arenas during the duration of the behavior testing. A camera was mounted above the arenas to record each trial. Each arena was thoroughly cleaned between trials with 70% ethanol, and fresh bedding was used to eliminate distinguishing olfactory cues (11-17). Data collections and analysis were done by two independent observers who were blinded to the identity of animal groups.

NOR: The NOR test was administered after 3 days of habituation (10 min/day). Objects to be distinguished were made of a similar plastic material but varied in color, shape, and size. To ensure that objects were in exactly the same position for each trial, magnets were used to hold them in place ≈ 7 cm from opposing corners and 16 cm apart. During the testing day, mice were

exposed to two duplicate objects within the arena for 5 min (familiarization). They were then allowed to rest for 5 min in their home cage within the behavioral room while the objects were cleaned (70% ethanol), and a novel object was added. Mice were then returned to the arena for 5 min, where they explored the familiar and novel object (testing). The objects were counterbalanced between the groups and these objects were not used in subsequent testing. Trials were later hand scored by an individual blinded to the experimental groups and calculated by using the discrimination index. A positive score is counted when the nose of the mouse is within 1 cm and pointed in the direction of the object. Time was not scored for mice that were near but not facing the object. To quantify preference or indifference for exploring novelty, a discrimination index was calculated as below. A positive score indicates a preference, or more time exploring the novel object, while a negative score indicates indifference, or more time exploring the familiar object. The novel object discrimination index was also directly compared between controls and irradiated mice. (1,2,9,10,12-14,18)

$$\left(\frac{\text{Time spent exploring novel object}}{\text{total exploration time}} - \frac{\text{Time spent exploring familiar object}}{\text{total exploration time}}\right) \times 100$$

OiP: The week after cessation of NOR testing, mice were habituated again to their arenas (10min/day) for 2 consecutive days. On the third day, mice were exposed to four objects of varying size, color, and shape for 5 min (familiarization). Mice were then allowed to rest for 5min in their home cage within the same room as the arenas. Objects were cleaned with 70% ethanol, and two of the four objects switched locations. The objects were counter balanced between the groups to assure there were no inherent preferences to a particular object. Mice were returned to the arenas for 5min of exploration (testing). Trials were later hand scored by an individual blinded to the experimental groups and calculated by using the discrimination index as described earlier. Positive scores were calculated based on the same criteria detailed for the NOR task earlier (1,2,9,10,12-14,18).

EPM: EPM is comprised of an acrylic surface with four elevated arms (75 cm above from the floor, 110 cm long and 10 cm wide) with two opposing arms closed with 42 cm high walls (i.e. closed arms). This defines the exploration of mice in 5 different zones: two open arm zones, two closed arm zones, and a central zone (measuring 10 x 10 cm area) where the arms intersect. Four weeks following irradiation each mouse was first placed in the central zone of the EPM with the head facing towards the closed arm. The mouse was allowed to freely explore the maze for 5 minutes and the entire test was done under dim light conditions. The frequency of entries and the amount of time spent in each arm was video recorded. Independent observers who were blinded to animal groups collected these data. At the end of 5 minutes, each mouse was removed from the EPM and placed back in its home cage. The EPM apparatus was thoroughly cleaned with Virkon® at 0.5-1 % solution before each trial to avoid any influence of odor related clues from the previous session. The percentage of open arm entries and duration of time spent in open arms were used to quantify anxiety-like behavior (13).

FST: FST is one of the most commonly used tasks for assessing depressive-like behavior in rodents. The test involves placing the mouse inside a cylinder filled with water, after which the mobility of the animal is measured for 6 to 10 minutes. Traditionally, ‘floating behavior’ (where the animal remains almost immobile and with its head above water) is used as a parameter to

analyze ‘hopelessness or despair’ and thus depression-like behavior. After the EPM test, each mouse was first placed in a glass beaker (having an inner diameter of 15 cm and depth of 20 cm) filled with tap water (20-22°C) to a depth of 16 cm. The depth of water used ensured that the animal could not touch the bottom of the container with their hind paws or tail. The FST was conducted in a single session comprising 6 minutes and the data were collected every minute for immobility or swimming during the test by two independent observers who were blind to the animal groups. Swimming in the FST is defined as the horizontal movement of the animal in the swim chamber and climbing refers to the vertically directed movement with forepaws mostly above the water along the wall of the swim chamber. Immobility or floating however is defined as the minimum movement necessary to keep the head above the water level. Mice were removed from the water at the end of 6 minutes and gently dried and placed back in their home cages. The total immobility time spent over the trial duration was calculated for every mouse and was used to quantify depression-like behavior (19).

Tissue processing and immunohistochemistry: Animals underwent intracardiac perfusions with 4% paraformaldehyde solution prepared in phosphate buffer. Each animal was first deeply anesthetized with isoflurane in a Plexiglas chamber until it ceased respiration. Following this, the chest was surgically opened, the heart was exposed, a nick was made in the right atrium, and sequentially infused with normal saline and 4% paraformaldehyde through the left ventricle. The brains were dissected, post-fixed overnight in 4% paraformaldehyde solution, washed in phosphate buffer, and treated with different concentrations (10–30%) of sucrose until they sank to the bottom of the container. Each brain was then mounted on a cryostat chuck and 30-micrometer thick coronal sections through the hippocampus were cut and collected serially in 24-well plates. Serial sections (every 10th) through the entire hippocampus were picked from each animal belonging to AM251(± 9Gy) and vehicle control(± 9Gy) groups and processed for BrdU and DCX immunostaining, as described in our previous studies (7,8,20,21). In order to measure the fraction of BrdU cells that express the immature neuronal marker (doublecortin, DCX) in the subgranular zone-granule cell layer (SGZ–GCL), another set of serial sections (every 10th) through the hippocampus was processed for BrdU and DCX dual immunofluorescence staining, as detailed previously (7). Furthermore, additional sets of serial sections (every 10th) through the hippocampus were processed for immunohistochemical characterization of cells positive for HMGB1. The primary antibodies comprised monoclonal antibodies against BrdU (MAB 3424, Millipore), NeuN (ABN78, Millipore), HMGB1 (Abcam, AB 18256), and polyclonal antibodies against DCX (C-8066; Santa Cruz Biotechnology).

Stereological quantification of BrdU, DCX neurons and NeuN+ cells: Numbers of BrdU+ newly born cells and DCX+ newly born neurons in the subgranular zone-granule cell layer (SGZ–GCL), and NeuN+ cell in SGZ-GCL and CA1, were stereologically measured using the optical fractionator method (n = 6 animals/group for BrdU and DCX, and n=4 for NeuN). For this, we used 30-µm thick serial sections (every 10th section) through the entire hippocampus and the StereoInvestigator system (Microbrightfield) comprising a color digital video interfaced with a Nikon microscope, as detailed in our earlier publications (3,14,20). In brief, the contour of the chosen counting region (SGZ-GCL for BrdU and DCX, and SGZ-GCL and CA1 for NeuN) was marked in every section, the optical fractionator component was activated, and the density and location of counting frames (each measuring 35 x 35 mm) and the counting depth were determined by entering parameters such as the grid size (75 mm), the thickness of top guard zone (2 mm) and the optical dissector height (i.e. 12 -14 mm). A computer driven motorized stage then allowed the cells to be measured at each of the counting frame locations. In every location,

the top of the section was set, and the plane of the focus was moved 4 mm deeper through the section (guard zone). This plane served as the first point of the counting process. All cells that came into focus in the next 12-14 mm section thickness were counted if they were entirely within the counting frame or touching the upper or right side of the counting frame. This procedure was repeated for all serial sections. By utilizing parameters such as the initial section thickness (i.e. at the time of sectioning), the section thickness at the time of cell counting (i.e. after immunostaining), and cell counts from the counting frame locations, the StereoInvestigator program calculated the total number of BrdU+, DCX+, or NeuN+ cells per SGZ-GCL or CA1, as described in our earlier studies. The Gundersen coefficient error (CE) was in the range of 0.05-0.15 for all counts in this (7,8,22)

Measurement of BrdU+ cells expressing the mature neuronal marker NeuN: The sections processed for BrdU and NeuN dual immunofluorescence were analyzed using a laser scanning confocal microscope (Nikon Eclipse TE2000-U, EZ-C1 interface, Nikon, Japan), as described in our earlier reports. The Z-stacks taken at 1-mmintervals were analyzed for identification of cells dual labeled for BrdU-and-NeuN using IMARIS volume tool (v7.6, Bitplane Inc., Switzerland). The minimum 50 BrdU-positive cells were counted for each animal, and net neurogenesis was calculated using total numbers of BrdU+ cells, and percentages of BrdU+ cells that differentiate into NeuN+ neurons(7,8,20,22).

Quantitative analyses of HMGB1 in hippocampus: For quantification of HMGB1, 3D deconvolution and reconstruction was carried out using the AutoQuantX3 algorithm (MediaCybernetics). Deconvolution combined with 3D reconstruction yields higher spatial resolution images for the immunofluorescent cell bodies. The Z-stacks taken in 1-µm intervals at at 60× were analyzed for quantification of HMGB1 expression, using IMARIS volume tool (v7.6, Bitplane Inc., Switzerland), that detects immunofluorescent cell bodies within 3D deconvoluted image stacks. Cell satisfying pre-defined criteria (verified visually for accuracy) were converted to surface for quantification under preset parameters kept constant throughout subsequent analyses. The “volume quality threshold” and “minimum surface volume” parameters were manually adjusted to optimize detection and kept constant thereafter for all subsequent analyses (1,12-15).

References:

1. Acharya MM, Green KN, Allen BD, Najafi AR, Syage A, Minasyan H, *et al.* Elimination of microglia improves cognitive function following cranial irradiation. Scientific reports **2016**;6:31545
2. Dey D, Parihar VK, Szabo GG, Klein PM, Tran J, Moayyad J, *et al.* Neurological Impairments in Mice Subjected to Irradiation and Chemotherapy. Radiation research **2020**;193:407-24
3. Baulch JE, Acharya MM, Allen BD, Ru N, Chmielewski NN, Martirosian V, *et al.* Cranial grafting of stem cell-derived microvesicles improves cognition and reduces neuropathology in the irradiated brain. Proceedings of the National Academy of Sciences of the United States of America **2016**;113:4836-41
4. Parihar VK, Limoli CL. Cranial irradiation compromises neuronal architecture in the hippocampus. Proceedings of the National Academy of Sciences of the United States of America **2013**;110:12822-7

5. Acharya MM, Christie LA, Hazel TG, Johe KK, Limoli CL. Transplantation of human fetal-derived neural stem cells improves cognitive function following cranial irradiation. Cell transplantation **2014**;23:1255-66
6. Jenrow KA, Brown SL, Liu J, Kolozsvary A, Lapanowski K, Kim JH. Ramipril mitigates radiation-induced impairment of neurogenesis in the rat dentate gyrus. Radiation oncology **2010**;5:6
7. Parihar VK, Hattiangady B, Kuruba R, Shuai B, Shetty AK. Predictable chronic mild stress improves mood, hippocampal neurogenesis and memory. Molecular psychiatry **2011**;16:171-83
8. Parihar VK, Hattiangady B, Shuai B, Shetty AK. Mood and memory deficits in a model of Gulf War illness are linked with reduced neurogenesis, partial neuron loss, and mild inflammation in the hippocampus. Neuropsychopharmacology : official publication of the American College of Neuropsychopharmacology **2013**;38:2348-62
9. Barker GR, Bird F, Alexander V, Warburton EC. Recognition memory for objects, place, and temporal order: a disconnection analysis of the role of the medial prefrontal cortex and perirhinal cortex. The Journal of neuroscience : the official journal of the Society for Neuroscience **2007**;27:2948-57
10. Barker GR, Warburton EC. Object-in-place associative recognition memory depends on glutamate receptor neurotransmission within two defined hippocampal-cortical circuits: a critical role for AMPA and NMDA receptors in the hippocampus, perirhinal, and prefrontal cortices. Cerebral cortex **2015**;25:472-81
11. Acharya MM, Baddour AA, Kawashita T, Allen BD, Syage AR, Nguyen TH, *et al.* Epigenetic determinants of space radiation-induced cognitive dysfunction. Scientific reports **2017**;7:42885
12. Parihar VK, Allen B, Tran KK, Macaraeg TG, Chu EM, Kwok SF, *et al.* What happens to your brain on the way to Mars. Science advances **2015**;1
13. Parihar VK, Allen BD, Caressi C, Kwok S, Chu E, Tran KK, *et al.* Cosmic radiation exposure and persistent cognitive dysfunction. Scientific reports **2016**;6:34774
14. Parihar VK, Allen BD, Tran KK, Chmielewski NN, Craver BM, Martirosian V, *et al.* Targeted overexpression of mitochondrial catalase prevents radiation-induced cognitive dysfunction. Antioxidants & redox signaling **2015**;22:78-91
15. Acharya MM, Baulch JE, Klein PM, Baddour AAD, Apodaca LA, Kramar EA, *et al.* New Concerns for Neurocognitive Function during Deep Space Exposures to Chronic, Low Dose-Rate, Neutron Radiation. eNeuro **2019**;6
16. Parihar VK, Maroso M, Syage A, Allen BD, Angulo MC, Soltesz I, *et al.* Persistent nature of alterations in cognition and neuronal circuit excitability after exposure to simulated cosmic radiation in mice. Experimental neurology **2018**;305:44-55
17. Acharya MM, Patel NH, Craver BM, Tran KK, Giedzinski E, Tseng BP, *et al.* Consequences of low dose ionizing radiation exposure on the hippocampal microenvironment. PloS one **2015**;10:e0128316
18. Barker GR, Warburton EC. When is the hippocampus involved in recognition memory? The Journal of neuroscience : the official journal of the Society for Neuroscience **2011**;31:10721-31
19. Slattery DA, Cryan JF. Using the rat forced swim test to assess antidepressant-like activity in rodents. Nat Protoc **2012**;7:1009-14

20. Acharya MM, Christie LA, Lan ML, Donovan PJ, Cotman CW, Fike JR, *et al.* Rescue of radiation-induced cognitive impairment through cranial transplantation of human embryonic stem cells. Proceedings of the National Academy of Sciences of the United States of America **2009**;106:19150-5
21. Parihar VK, Acharya MM, Roa DE, Bosch O, Christie LA, Limoli CL. Defining functional changes in the brain caused by targeted stereotaxic radiosurgery. Translational cancer research **2014**;3:124-37
22. Dey D, Parihar VK, Szabo GG, Klein PM, Tran J, Moayyad J, *et al.* Neurological Impairments in Mice Subjected to Irradiation and Chemotherapy. Radiation research **2020**
